# Serum FGF-23 and α-Klotho Levels in Prolactinoma and Non-Tumoral Hyperprolactinemia: An Exploratory Cross-Sectional Comparative Study

**DOI:** 10.3390/diagnostics16142298

**Published:** 2026-07-22

**Authors:** Abdullah Altuğ, Fatma Zehra Yıldırım Arı, Mithat Mızrak, Mehmet Ferit Gürsu, Tuğçe Kaymaz, Emir Dönder, Ahmet Yılmaz

**Affiliations:** 1 Department of Internal Medicine, Faculty of Medicine, Fırat University, Elazığ 23119, Türkiye; e.donder33@hotmail.com; 2 Department of Biochemistry, Faculty of Medicine, Fırat University, Elazığ 23119, Türkiye; yildirimfatmazehra@gmail.com (F.Z.Y.A.); fegursu@yahoo.com (M.F.G.); tugcekaymaz.92@gmail.com (T.K.); emir_949_@hotmail.com (A.Y.); 3 Department of Endocrinology, Faculty of Medicine, Fırat University, Elazığ 23119, Türkiye; mithat.mizrak@gmail.com

**Keywords:** prolactinoma, non-tumoral hyperprolactinemia, FGF-23, α-Klotho, dopamine agonist, ROC analysis

## Abstract

**Background/Objectives:** This study aimed to evaluate serum fibroblast growth factor-23 (FGF-23) and α-Klotho levels in patients with prolactinoma and patients with non-tumoral hyperprolactinemia and to explore their discriminatory performance for prolactinoma. **Methods:** Seventy-three participants were included in this single-center, cross-sectional comparative study: non-tumoral hyperprolactinemia (*n* = 14), prolactinoma (*n* = 29), and healthy controls (*n* = 30). All patients with prolactinoma had received dopamine agonist therapy for at least six months. Serum FGF-23 and α-Klotho were measured by ELISA, and between-group comparisons, correlation analyses, linear regression analyses, and receiver operating characteristic (ROC) analyses were performed. **Results:** FGF-23 concentrations differed significantly among the groups (overall *p* < 0.001). Post hoc analysis showed that FGF-23 concentrations were significantly higher in the prolactinoma group [236.10 (107.70–667.90) pg/mL] than in healthy controls [138.75 (49.40–253.60) pg/mL] (adjusted *p* < 0.001), whereas the difference between the prolactinoma and non-tumoral hyperprolactinemia groups [166.20 (100.30–327.20) pg/mL] was not statistically significant (adjusted *p* = 0.075). α-Klotho concentrations were significantly higher in the prolactinoma group [2.908 (1.376–6.257) ng/mL] than in both the non-tumoral hyperprolactinemia group [2.244 (1.432–5.139) ng/mL] (adjusted *p* = 0.038) and healthy controls [2.204 (0.149–4.917) ng/mL] (adjusted *p* < 0.001). In pooled analyses, both biomarkers showed positive correlations with prolactin and weak negative correlations with hemoglobin. However, prolactin and hemoglobin were not independently associated with either biomarker after multivariable adjustment. The non-tumoral hyperprolactinemia group did not differ significantly from healthy controls for either biomarker. For distinguishing prolactinoma from non-tumoral hyperprolactinemia, the AUCs were 0.741 for FGF-23, 0.732 for α-Klotho, and 0.862 for the exploratory combined model. **Conclusions:** Dopamine agonist-treated prolactinoma was characterized by higher circulating FGF-23 concentrations than those in healthy controls and by higher α-Klotho concentrations than those in both healthy controls and patients with non-tumoral hyperprolactinemia. Exploratory ROC analyses suggested moderate discriminatory performance for the individual biomarkers and higher apparent performance for the combined model; however, these findings should be interpreted cautiously and require validation in larger, treatment-naïve cohorts.

## 1. Introduction

Prolactinoma is one of the most common functional neuroendocrine tumors of the pituitary gland and accounts for approximately 40% of pituitary adenomas. Its prevalence has been estimated at approximately 50 per 100,000 individuals, with a predominance among women of reproductive age [1,2]. Clinical manifestations include symptoms resulting from hyperprolactinemia, such as amenorrhea, galactorrhea, infertility, hypogonadism, and sexual dysfunction, as well as headache, visual field defects, and hypopituitarism caused by the tumor mass effect, particularly in patients with macroadenomas [2].

The clinical consequences of prolactinoma and chronic hyperprolactinemia extend beyond the reproductive axis. Prolactin excess and the associated hypogonadism may affect bone turnover, bone mineral density, body composition, insulin sensitivity, lipid metabolism, and cardiometabolic health. Persistent bone impairment has been reported in patients with prolactinoma despite long-term control of hyperprolactinemia and hypogonadism [3]. In addition, dopamine agonist therapy may improve insulin sensitivity, glucose metabolism, and lipid profiles [4,5]. Prolactinoma should therefore be considered a systemic endocrine disorder with potentially important bone and metabolic consequences.

Fibroblast growth factor-23 (FGF-23) is a bone-derived endocrine factor produced mainly by osteocytes and osteoblasts. It promotes renal phosphate excretion and suppresses the synthesis of 1,25-dihydroxyvitamin D, thereby playing a central role in phosphate and vitamin D homeostasis [6,7]. Because hyperprolactinemia-associated hypogonadism may alter bone turnover and bone mineral density, the evaluation of circulating FGF-23 in patients with prolactinoma is biologically relevant.

α-Klotho acts as an essential co-receptor for fibroblast growth factor receptors in classical FGF-23 signaling [8,9,10]. However, its biological functions extend beyond mineral metabolism and include the regulation of oxidative stress, inflammation, insulin/IGF-1 signaling, aging-related processes, and metabolic homeostasis [10,11,12,13,14]. Experimental evidence also suggests that α-Klotho may modulate oxidative stress responses associated with dopaminergic signaling [15]. Because dopamine agonists constitute the main pharmacological treatment for prolactinoma, this potential relationship provides an additional rationale for evaluating circulating α-Klotho in this clinical setting.

Previous studies have evaluated FGF-23 or α-Klotho in patients with prolactinoma or hyperprolactinemia; however, the FGF-23/α-Klotho axis has not been sufficiently investigated using non-tumoral hyperprolactinemia as a separate comparator group. Such a comparison may help determine whether alterations in these biomarkers are associated primarily with prolactin elevation or with a broader biochemical pattern related to prolactinoma, its systemic consequences, or dopamine agonist exposure.

Accordingly, this study aimed to compare serum FGF-23 and α-Klotho concentrations among patients with dopamine agonist-treated prolactinoma, patients with non-tumoral hyperprolactinemia, and healthy controls. We also evaluated the relationships between these biomarkers and prolactin and selected biochemical parameters, assessed their independent associations using multivariable regression analyses, and explored their individual and combined discriminatory performance for distinguishing prolactinoma from non-tumoral hyperprolactinemia and healthy controls.

## 2. Materials and Methods

### 2.1. Research Ethics

This study was designed as a single-center, cross-sectional, comparative clinical study. The study was approved by the Ethics Committee of Fırat University with decision number 2025/02-46, dated 30 January 2025. Written informed consent was obtained from all participants before enrollment.

### 2.2. Participants and Study Groups

A total of 73 participants aged 17–65 years were included in the study. Participants were divided into three groups: the prolactinoma group, the non-tumoral hyperprolactinemia group, and the healthy control group.

The prolactinoma group consisted of 29 patients diagnosed with prolactinoma according to established clinical, biochemical, and pituitary magnetic resonance imaging criteria recommended by the Pituitary Society International Consensus Statement [2]. All patients in this group were receiving dopamine agonist therapy at the time of the study and had been using cabergoline and/or bromocriptine for at least 6 months.

The non-tumoral hyperprolactinemia group consisted of 14 patients with persistent hyperprolactinemia but without radiological evidence of prolactinoma on pituitary magnetic resonance imaging. Eligible participants were consecutively recruited from the Endocrinology Outpatient Clinic during the study period according to predefined inclusion and exclusion criteria. Macroprolactinemia was excluded before group allocation using polyethylene glycol (PEG) precipitation according to routine laboratory practice [16]. Common secondary causes of hyperprolactinemia, including pregnancy or lactation, primary hypothyroidism, clinically significant renal dysfunction, and the use of medications known to increase prolactin levels, were excluded through clinical and laboratory evaluation. Patients without an identifiable pituitary lesion or another common secondary cause of hyperprolactinemia were classified as having non-tumoral hyperprolactinemia.

The control group consisted of 30 healthy volunteers with no known endocrine or systemic disease. The diagnosis of prolactinoma and treatment history were confirmed by reviewing the patients’ medical records at the time of study enrollment.

### 2.3. Exclusion Criteria

Known renal failure, active malignancy, chronic inflammatory disease, pregnancy, lactation, and the use of medications other than prescribed dopamine agonist therapy that could affect phosphate–mineral metabolism were considered exclusion criteria.

### 2.4. Blood Samples and Biomarker Measurements

Following at least 12 h of overnight fasting, venous blood samples were collected at 08:00 a.m. to minimize the influence of the known circadian variation in FGF-23. Samples were kept at room temperature for 20 min and then centrifuged at 3000 rpm for 10 min. Serum was separated and stored at −20 °C until analysis.

Routine biochemical parameters were obtained from thbe outpatient clinic laboratory records. Aspartate aminotransferase (AST), alanine aminotransferase (ALT), alkaline phosphatase (ALP), gamma-glutamyl transferase (GGT), creatine kinase (CK), CK-MB, lactate dehydrogenase (LDH), total cholesterol, high-density lipoprotein (HDL), low-density lipoprotein (LDL), C-reactive protein (CRP), calcium, phosphorus, urea, uric acid, creatinine, total protein, albumin, and total bilirubin were measured using commercial reagents on a Siemens ADVIA Chemistry XPT System (Siemens Healthineers AG, Erlangen, Germany). White blood cell count (WBC) and hemoglobin measurements were performed using a Sysmex XN-1000 automated hematology analyzer (Sysmex Corporation, Kobe, Japan).

Serum FGF-23 and α-Klotho concentrations were measured using sandwich enzyme-linked immunosorbent assay (ELISA) kits (Shanghai Sunred Biological Technology Co., Ltd., Shanghai, China). FGF-23 concentrations were determined using a Sunredbio Human FGF-23 ELISA kit (Cat. No. 201-12-0060; Shanghai Sunred Biological Technology Co., Ltd., Shanghai, China), whereas α-Klotho concentrations were measured using a Sunredbio Human KLOTHO ELISA kit (Cat. No. 201-12-2782; Shanghai Sunred Biological Technology Co., Ltd., Shanghai, China). The measurement ranges were 10–1500 pg/mL for FGF-23 and 0.1–20 ng/mL for α-Klotho, with analytical sensitivities of 5.147 pg/mL and 0.05 ng/mL, respectively. According to the manufacturer, the intra-assay coefficient of variation was <10%, and the inter-assay coefficient of variation was <12% for both assays. Serum FGF-23 and α-Klotho concentrations were expressed as pg/mL and ng/mL, respectively. All ELISA measurements were performed in singlet according to the manufacturer’s instructions. The microplates were washed using a BioTek ELx50 Microplate Washer (BioTek Instruments, Winooski, VT, USA), and optical density was measured at 450 nm using an Agilent BioTek Epoch 2 Microplate Reader (Agilent Technologies, Santa Clara, CA, USA) with BioTek Gen5 software (version 3.05).

### 2.5. Statistical Analysis

Statistical analyses were performed using IBM SPSS Statistics for Windows, version 27.0 (IBM Corp., Armonk, NY, USA). Dunn–Bonferroni post hoc pairwise comparisons were performed using jamovi, version 2.7.2. Data distribution was assessed using the Shapiro–Wilk test. Continuous variables with a normal distribution are presented as the mean ± standard deviation (SD), whereas non-normally distributed variables are presented as median (minimum–maximum). Between-group comparisons were performed using one-way analysis of variance (ANOVA) for normally distributed variables or the Kruskal–Wallis test for non-normally distributed variables. When the overall Kruskal–Wallis test was significant, Dunn–Bonferroni post hoc pairwise comparisons were performed to identify differences between groups while controlling the family-wise error rate. Categorical variables were analyzed using the Pearson chi-square test, Fisher’s exact test, or the Fisher–Freeman–Halton exact test, as appropriate. Correlations between variables were evaluated using Spearman’s rank correlation coefficient. To identify independent predictors of serum FGF-23 and α-Klotho concentrations, univariable and multivariable linear regression analyses were performed using log-transformed FGF-23 and α-Klotho values as the dependent variables. Variables were selected based on their clinical relevance and entered simultaneously into the multivariable models. The diagnostic performance of FGF-23 and α-Klotho was evaluated using receiver operating characteristic (ROC) curve analysis, and the combined model was internally validated using leave-one-out cross-validation (LOOCV). Optimal cut-off values for ROC analyses were determined using the maximum Youden index. Statistical significance was defined as a two-tailed *p* value < 0.05. Because no a priori power analysis was performed before data collection, a sensitivity analysis was conducted using G*Power software (version 3.1.9.7) to evaluate the statistical sensitivity of the multivariable linear regression model (F tests, linear multiple regression: fixed model, R^2^ deviation from zero). Based on a total sample size of 73 participants, six predictors, a significance level of α = 0.05, and 80% statistical power (1 − β = 0.80), the study was able to detect effect sizes of f^2^ = 0.205 or greater.

## 3. Results

### 3.1. Demographic and Clinical Characteristics

A total of 73 participants were included in the study. Fourteen participants were in the non-tumoral hyperprolactinemia group, 29 were in the prolactinoma group, and 30 were in the healthy control group. No significant difference was found among the groups in terms of sex distribution; the proportion of women was 92.9% in the non-tumoral hyperprolactinemia group, 72.4% in the prolactinoma group, and 66.7% in the control group (*p* = 0.177). Smoking status also did not differ significantly among the groups (*p* = 0.655).

There was a significant difference among the groups in terms of age (*p* < 0.001). The median age was 24.5 (19–50) years in the non-tumoral hyperprolactinemia group, 37.0 (20–60) years in the prolactinoma group, and 22.5 (18–59) years in the healthy control group. Dunn–Bonferroni post hoc analysis demonstrated that the prolactinoma group was significantly older than both the healthy control group (adjusted *p* < 0.001) and the non-tumoral hyperprolactinemia group (adjusted *p* = 0.043), whereas no significant difference was observed between the non-tumoral hyperprolactinemia and healthy control groups (adjusted *p* = 1.000). No significant difference was observed among the groups in terms of body mass index; the median BMI values were 22.43, 25.71, and 23.61 kg/m^2^ in the non-tumoral hyperprolactinemia, prolactinoma, and healthy control groups, respectively (*p* = 0.080) (Table 1).

### 3.2. Routine Biochemical Parameters

When routine biochemical and hematological parameters were evaluated, no significant differences were observed among the groups in glucose, liver enzymes, lipid parameters, CRP, calcium, phosphorus, urea, creatinine, uric acid, total protein, albumin, and total bilirubin levels.

Hemoglobin levels did not differ significantly among the three groups (one-way ANOVA, F(2,70) = 2.732, *p* = 0.072) (Table 2).

### 3.3. FGF-23, α-Klotho, and Prolactin Levels

FGF-23 levels differed significantly among the groups (Kruskal–Wallis test, *p* < 0.001). Dunn–Bonferroni post hoc comparisons showed that the prolactinoma group had significantly higher FGF-23 levels than the healthy control group (adjusted *p* < 0.001). No significant differences were observed between the prolactinoma and non-tumoral hyperprolactinemia groups (adjusted *p* = 0.075) or between the non-tumoral hyperprolactinemia and healthy control groups (adjusted *p* = 0.466).

To evaluate the potential influence of sex on circulating FGF-23 concentrations, an additional subgroup analysis was performed using the Mann–Whitney U test. No significant difference in circulating FGF-23 concentrations was observed between female and male participants (Mann–Whitney U = 464.0, Z = −0.616, *p* = 0.538).

A significant difference was also detected among the groups in terms of α-Klotho levels (Kruskal–Wallis χ^2^ = 14.2, *p* < 0.001). Dunn–Bonferroni post hoc pairwise comparisons showed that α-Klotho levels were significantly higher in the prolactinoma group [2.908 (1.376–6.257) ng/mL] than in the non-tumoral hyperprolactinemia group [2.244 (1.432–5.139) ng/mL] (adjusted *p* = 0.038) and the healthy control group [2.204 (0.149–4.917) ng/mL] (adjusted *p* < 0.001). No significant difference was observed between the non-tumoral hyperprolactinemia and healthy control groups (adjusted *p* = 1.000) (Table 3).

### 3.4. Correlation Analyses

The relationships between FGF-23 and α-Klotho levels and biochemical parameters were evaluated using Spearman’s correlation analysis. A significant positive correlation was found between FGF-23 and prolactin (*r_s_* = 0.481, *p* < 0.001). A significant positive correlation was also observed between α-Klotho and prolactin (*r_s_* = 0.269, *p* = 0.021).

**Figure 1:**
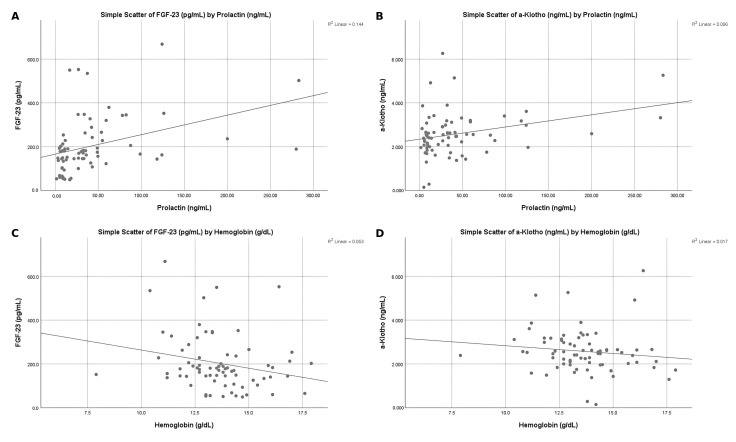
Scatter plots illustrating the relationships between circulating biomarkers and clinical parameters. (**A**) FGF-23 versus prolactin, (**B**) α-Klotho versus prolactin, (**C**) FGF-23 versus hemoglobin, and (**D**) α-Klotho versus hemoglobin. Solid lines represent the fitted linear regression lines.

Both biomarkers were also significantly negatively correlated with hemoglobin. The correlation between FGF-23 and hemoglobin was *r_s_* = −0.257 (*p* = 0.028), whereas the correlation between α-Klotho and hemoglobin was *r_s_* = −0.234 (*p* = 0.046). A positive but non-significant correlation was observed between FGF-23 and α-Klotho (*r_s_* = 0.210, *p* = 0.074). Selected metabolic, mineral, and renal parameters, including glucose, calcium, phosphorus, urea, and creatinine, are presented in Table 4; none showed significant correlations with either FGF-23 or α-Klotho. The significant correlations of circulating FGF-23 and α-Klotho concentrations with prolactin and hemoglobin are illustrated in Figure 1.

### 3.5. Linear Regression Analysis

To identify independent predictors of serum FGF-23 and α-Klotho concentrations, univariable and multivariable linear regression analyses were performed using log-transformed FGF-23 and α-Klotho values as the dependent variables.

For log-transformed FGF-23, univariable analyses demonstrated that prolactinoma status (B = 0.290, β = 0.542, 95% CI: 0.183–0.396, *p* < 0.001), serum prolactin concentration (B = 0.002, β = 0.386, 95% CI: 0.001–0.003, *p* = 0.001), and hemoglobin level (B = −0.036, β = −0.242, 95% CI: −0.070 to −0.002, *p* = 0.039) were significantly associated with log-transformed FGF-23 concentrations. In contrast, age and sex were not significantly associated with circulating FGF-23 concentrations in the univariable analyses (*p* = 0.058 and *p* = 0.835, respectively). The overall multivariable regression model was statistically significant (R^2^ = 0.391, adjusted R^2^ = 0.336, F(6,66) = 7.069, *p* < 0.001). After adjustment for age, sex, prolactin, and hemoglobin, both non-tumoral hyperprolactinemia (B = 0.179, 95% CI: 0.031–0.327, *p* = 0.018) and prolactinoma (B = 0.331, 95% CI: 0.188–0.474, *p* < 0.001) remained independently associated with higher circulating FGF-23 concentrations. In contrast, age, sex, serum prolactin concentration, and hemoglobin were not independently associated with circulating FGF-23 concentrations (Table 5).

For log-transformed α-Klotho, univariable analyses demonstrated significant associations with prolactinoma status and serum prolactin concentration. However, the multivariable regression model was not statistically significant (R^2^ = 0.136, adjusted R^2^ = 0.057, F(6,66) = 1.730, *p* = 0.128), and none of the evaluated variables remained independently associated with circulating α-Klotho concentrations after adjustment (Table 6).

### 3.6. ROC Analysis

The performance of FGF-23 and α-Klotho levels in distinguishing the prolactinoma group from the non-tumoral hyperprolactinemia group and the healthy control group was evaluated using ROC analysis.

In the comparison between the prolactinoma and non-tumoral hyperprolactinemia groups, the area under the curve for FGF-23 was 0.741 (95% CI: 0.577–0.882; *p* = 0.012). At the optimal cut-off value of 183.40 pg/mL, the sensitivity and specificity of FGF-23 were 69.0% and 78.6%, respectively. For α-Klotho, the area under the curve was 0.732 (95% CI: 0.540–0.893; *p* = 0.015). At the cut-off value of 2.28 ng/mL, the sensitivity and specificity of α-Klotho were 89.7% and 57.1%, respectively.

In the comparison between the prolactinoma group and healthy controls, the area under the curve for FGF-23 in distinguishing the prolactinoma group from healthy controls was 0.832 (95% CI: 0.728–0.922; *p* < 0.001). At the optimal cut-off value of 205.70 pg/mL, the sensitivity and specificity of FGF-23 were 58.6% and 90.0%, respectively. For α-Klotho, the area under the curve was 0.774 (95% CI: 0.638–0.891; *p* < 0.001). At the cut-off value of 2.41 ng/mL, the sensitivity and specificity of α-Klotho were 86.2% and 66.7%, respectively.

**Figure 2:**
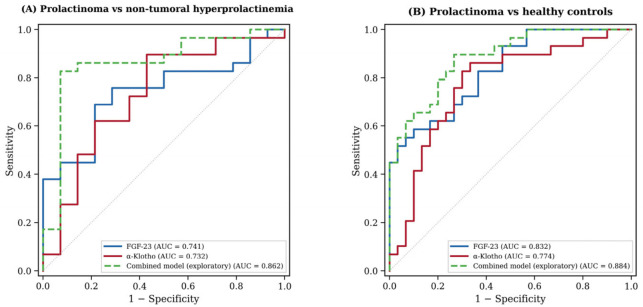
Receiver operating characteristic (ROC) curves of FGF-23, α-Klotho, and the exploratory combined model for distinguishing prolactinoma from non-tumoral hyperprolactinemia (**A**) and healthy controls (**B**). The combined model was generated using an exploratory logistic regression model including both FGF-23 and α-Klotho.

In the exploratory logistic regression model including both FGF-23 and α-Klotho, discriminatory performance was higher than that of the individual biomarkers. The area under the curve of the combined model was 0.862 (95% CI: 0.641–0.974) for distinguishing prolactinoma from non-tumoral hyperprolactinemia and 0.884 (95% CI: 0.796–0.960) for distinguishing prolactinoma from healthy controls. In the internal validation performed using LOOCV, the cross-validated AUC of the combined model was 0.764 for the prolactinoma–non-tumoral hyperprolactinemia comparison and 0.836 for the prolactinoma–healthy control comparison (Table 7, Figure 2).

## 4. Discussion

The principal finding of this study was a distinct biomarker pattern across the three study groups. FGF-23 concentrations were significantly higher in patients with prolactinoma than in healthy controls, whereas the difference between the prolactinoma and non-tumoral hyperprolactinemia groups was not statistically significant after adjustment for multiple comparisons. In contrast, α-Klotho concentrations were significantly higher in the prolactinoma group than in both comparator groups. Because prolactin concentrations were elevated in both patient groups, these findings suggest that prolactin excess alone is unlikely to fully explain the observed biomarker profile. Instead, the observed differences may reflect prolactinoma-related bone metabolism, dopamine agonist exposure, or a combination of these factors.

Although prolactinoma and hyperprolactinemia are classically defined by their effects on the reproductive axis, current evidence indicates that these conditions also have important effects on bone mineral density, insulin sensitivity, lipid profiles, and cardiometabolic balance [3,4,5]. FGF-23 is an osteocyte- and osteoblast-derived endocrine factor that plays a fundamental role in the regulation of phosphate excretion and vitamin D metabolism [6,7]. Therefore, evaluating FGF-23 levels in the context of hyperprolactinemia-related hypogonadism and bone turnover alterations is biologically meaningful. The higher FGF-23 concentrations observed in prolactinoma than in healthy controls may reflect a more pronounced bone–metabolic profile; however, the absence of a significant direct difference from non-tumoral hyperprolactinemia requires cautious interpretation.

The principal study in the literature evaluating FGF-23 levels in patients with prolactinoma was conducted by Arslan MS et al. [17]. In that cross-sectional study, 46 premenopausal women with prolactinoma were divided into two subgroups, newly diagnosed patients and patients receiving cabergoline treatment, and were compared with 20 healthy controls. The authors examined FGF-23, OPG, RANKL, vitamin D, calcium, phosphorus, and PTH levels and reported that FGF-23 levels were similar among the groups [17]. By contrast, in our study, FGF-23 concentrations were significantly higher in the prolactinoma group than in healthy controls. Although median concentrations were also higher than those in the non-tumoral hyperprolactinemia group, the direct between-group difference was not statistically significant. Possible reasons for this difference may include differences in the study population, sex distribution, treatment status, measurement method, and comparator groups. Whereas the study by Arslan MS et al. investigated the role of the FGF-23/OPG/RANKL system in hyperprolactinemia-related bone loss, our study evaluated FGF-23 together with α-Klotho and within the context of differentiating prolactinoma from non-tumoral hyperprolactinemia. This distinction suggests that FGF-23 in the context of prolactinoma should be considered not only through classical bone mineral metabolism but also within a broader tumor–metabolism response. Notably, the absence of significant differences in calcium and phosphorus levels among the groups in the present cohort suggests that the increase in FGF-23 and serum α-Klotho may not simply reflect an overt calcium–phosphorus imbalance, but rather a more complex bone–metabolic or adaptive response associated with prolactinoma.

In addition to being an important co-receptor for classical phosphate and vitamin D metabolism in FGF-23 signaling, α-Klotho is a multifunctional protein involved in oxidative stress, inflammation, cellular aging, insulin/IGF-1 signaling, and metabolic homeostasis [8,9,10,11]. The development of aging-like phenotypes in mice with *klotho* gene deficiency supports the fundamental role of Klotho in aging biology [12]. Human studies have reported that serum-measured sαKL levels may show age-related changes [13,14]. In our study, the age distribution differed significantly among the groups, and the median age was highest in the prolactinoma group. Nevertheless, serum α-Klotho levels were also elevated in this group, which is noteworthy. Considering that α-Klotho is related to aging biology and may decrease with age, the finding of elevated serum α-Klotho levels in the prolactinoma group with the highest median age does not appear to be a finding that can be explained by a simple age effect. On the contrary, this finding suggests that a distinct biological response related to α-Klotho-associated aging biology, oxidative stress balance, metabolic adaptation, or dopamine agonist therapy may be present in the prolactinoma group. Schweizer et al. reported that sαKL levels were slightly higher in women than in men in the reference population [18]. In our study, although sex distribution did not differ significantly among the groups (*p* = 0.177), the proportion of men in the prolactinoma group was relatively higher than that in the non-tumoral hyperprolactinemia group. Considering that sαKL levels may be higher in women, the finding of elevated serum α-Klotho levels in the prolactinoma group makes it difficult to attribute this difference solely to sex distribution.

One of the most recent clinical data sets supporting increased sαKL in the context of prolactinoma was reported in the 2025 study by Schweizer et al. [18]. In that study, reference intervals for sαKL were established in a large reference population of 890 adults; sαKL levels were also evaluated in patients with non-functioning pituitary adenoma and prolactinoma. The authors reported that sαKL levels in patients with NFPA did not differ from those in the reference population, whereas sαKL levels were mildly but significantly increased in patients with prolactinoma [18]. This finding is consistent with the elevated serum α-Klotho levels observed in the prolactinoma group in our study. A key limitation of the study by Schweizer et al. was the absence of a non-tumoral hyperprolactinemia group; therefore, it was not possible to clearly distinguish whether the sαKL elevation in the prolactinoma group was related to prolactin elevation, tumor presence, demographic characteristics, or treatment-related factors. The distinctive aspect of our study is that it also included a non-tumoral hyperprolactinemia group, thereby allowing a clearer separation between prolactin elevation and the presence of prolactinoma. The fact that the non-tumoral hyperprolactinemia group did not differ from the control group in terms of serum α-Klotho levels supports the interpretation that this increase may not be explained solely by prolactin elevation.

In terms of the relationship between hyperprolactinemia and serum α-Klotho, the study by Arslan IE et al. also provides background supporting our results [19]. In that study, 33 women with hyperprolactinemia and 28 healthy controls were evaluated, and although prolactin levels were markedly higher in the hyperprolactinemia group, serum α-Klotho levels did not differ significantly between the groups [19]. In addition, cabergoline or bromocriptine use within the previous year was excluded in that study. In this respect, the absence of a marked increase in serum α-Klotho levels in the presence of hyperprolactinemia independent of the dopamine agonist effect strengthens the interpretation that the serum α-Klotho elevation detected in our study may not be explained solely by prolactin elevation. Nevertheless, in the hyperprolactinemia group of Arslan IE et al., cases with microadenoma and macroadenoma detected on pituitary magnetic resonance imaging were also included. In our study, however, the hyperprolactinemia group consisted of patients without prolactinoma on pituitary MRI. This distinction is an important point added by our study to the literature because it evaluates prolactin elevation and the presence of prolactinoma as separate clinical groups rather than under a single category.

Differences in ELISA kits used for the interpretation of serum α-Klotho measurements should also be considered. Schweizer et al. emphasized that different commercial α-Klotho ELISA systems may yield markedly different absolute concentrations in the same samples and that direct comparison of results obtained only with the same measurement system is more appropriate [18]. In our study, the SUNRED Human KLOTHO ELISA kit was used, whereas Schweizer et al. used the IBL sαKL ELISA system. Therefore, direct numerical comparison of absolute serum α-Klotho and sαKL values is not appropriate; nevertheless, the observation of a similar circulating α-Klotho increase pattern in the prolactinoma group across different kits and different populations suggests that this finding may not be merely a random result related to the measurement system.

Although these limitations related to measurement methods should be taken into account, the differing between-group and adjusted patterns of FGF-23 and α-Klotho suggest that these biomarkers may be influenced by partially distinct biological processes in the context of prolactinoma. First, chronic hyperprolactinemia and hypogonadism accompanying prolactinoma may disrupt bone turnover, and persistent hyperprolactinemia has been shown to be associated with long-term loss of bone mineral density [3]. Because FGF-23 is produced mainly by osteocytes and osteoblasts, alterations in bone turnover may affect FGF-23 synthesis [6,7]. Second, prolactinoma and hyperprolactinemia may also affect insulin sensitivity, lipid profiles, and cardiometabolic balance [4,5]. α-Klotho is considered a multifunctional protein associated with insulin/IGF-1 signaling and metabolic homeostasis [10,11]. Therefore, elevated α-Klotho in the prolactinoma group may also be interpreted as an adaptive response to metabolic or oxidative stress accompanying tumor presence.

This possible mechanistic divergence is also partly reflected in the correlation analyses. In our study, a moderate positive correlation was found between FGF-23 and prolactin, whereas a weaker but significant positive correlation was observed between α-Klotho and prolactin. In contrast, the relationship between FGF-23 and α-Klotho was positive but did not reach statistical significance. This distribution suggests that FGF-23 and α-Klotho may be related to different but partially complementary processes rather than reflecting exactly the same biological process in the context of prolactinoma. Notably, in the study by Schweizer et al., a weak positive correlation between sαKL and prolactin was also reported in a subgroup of the reference population (*r_s_* = 0.31) [18]. In our study, a relationship in the same direction and of similar magnitude was found between serum α-Klotho and prolactin (*r_s_* = 0.269). Although this finding suggests a possible biological relationship between prolactin and α-Klotho, the absence of a difference in serum α-Klotho levels between the non-tumoral hyperprolactinemia and control groups in our study indicates that this relationship cannot be explained by prolactin level alone.

In pooled analyses, both biomarkers showed weak negative correlations with hemoglobin. Hemoglobin was also negatively associated with FGF-23 in the univariable regression model, although it was not an independent predictor after multivariable adjustment; no independent association was observed for α-Klotho. Hemoglobin concentrations did not differ significantly among the study groups. Hypogonadism-related suppression of erythropoiesis provides a biologically plausible context for these findings, as suggested by Rudman et al. [20]. Nevertheless, the cross-sectional design and the absence of comprehensive gonadal hormone and iron-status measurements preclude causal inference. These associations should therefore be considered hypothesis-generating, and future studies incorporating iron parameters, gonadal hormone profiles, inflammatory markers, and longitudinal biomarker measurements are needed to clarify their biological significance.

Age was another potential confounding factor, as the prolactinoma group had the highest median age among the study groups. However, this possibility was specifically addressed in our regression analyses. Age was not significantly associated with circulating FGF-23 concentrations in either the univariable or multivariable regression models, indicating that age was not an independent determinant of this biomarker. Therefore, the elevated circulating FGF-23 concentrations observed in patients with prolactinoma are unlikely to be explained solely by differences in age. Instead, these findings suggest that factors other than chronological age are more likely to contribute to the elevated circulating FGF-23 concentrations observed in patients with prolactinoma.

The fact that all patients in the prolactinoma group had been receiving dopamine agonist therapy for at least six months is critically important for interpreting serum α-Klotho elevation. While the non-tumoral hyperprolactinemia group did not use dopamine agonists, the prolactinoma group was receiving cabergoline and/or bromocriptine. This treatment difference raises the possibility that serum α-Klotho elevation may reflect not only tumor biology but also an adaptive response related to pharmacological modulation of dopaminergic signaling. Brobey et al. showed that Klotho overexpression exerted a protective effect against MPTP-induced dopaminergic neuronal injury in a mouse model and that this effect may be associated with modulation of the ASK1/p38 MAPK stress signaling pathway [15]. Experimental data regarding cabergoline may also support this interpretation. Odaka et al. reported that cabergoline could reduce neuronal cell death under oxidative stress and that this effect might be related to dopamine D2 receptor-mediated mechanisms [21]. Therefore, the possibility that serum α-Klotho elevation may have developed secondary to dopamine agonist therapy does not reduce the biological importance of the finding; rather, it generates a new hypothesis worth investigating regarding the relationship among dopaminergic therapy, oxidative stress, metabolic adaptation, and the α-Klotho axis. Given the cross-sectional design of the present study, the direction of this relationship cannot be determined. Therefore, it is not appropriate to attribute serum α-Klotho elevation in the prolactinoma group solely to tumor presence or solely to a treatment effect.

Another possible explanation raised in the study by Schweizer et al. should also be considered. The authors noted that most patients with prolactinoma had not undergone surgery and stated that co-expression of prolactin and growth hormone could not be excluded [18]. Considering that sαKL is markedly elevated in active acromegaly and is regarded as a GH-sensitive marker, a possible subclinical GH/IGF-1 axis effect may also have contributed to serum α-Klotho elevation in patients with prolactinoma. Because GH and IGF-1 levels were not measured in our study, this alternative explanation could not be directly evaluated. Simultaneous evaluation of the GH/IGF-1 axis in patients with prolactinoma in future studies may contribute to a better understanding of the source of α-Klotho elevation.

ROC analyses indicated moderate exploratory discriminatory performance for both FGF-23 and α-Klotho in distinguishing prolactinoma from non-tumoral hyperprolactinemia. For FGF-23, this discriminatory signal was observed despite the absence of a statistically significant direct pairwise difference between the two groups. Because group comparisons and ROC analyses address different statistical questions, these findings should be interpreted as complementary but exploratory. FGF-23 provided a more balanced and relatively specific profile, whereas α-Klotho showed higher sensitivity but lower specificity. This different sensitivity–specificity profile suggests that the two markers may reflect different biological processes in the context of prolactinoma. Indeed, discriminatory performance increased compared with that of individual markers in the exploratory logistic regression model including both FGF-23 and α-Klotho. These findings suggest that the combined evaluation of FGF-23 and α-Klotho may improve discrimination between prolactinoma and non-tumoral hyperprolactinemia. However, given the exploratory nature of the model and the limited sample size, this observation requires external validation in larger treatment-naïve cohorts before any clinical application can be considered.

The multivariable analyses showed that both non-tumoral hyperprolactinemia and prolactinoma status were independently associated with higher FGF-23 concentrations compared with healthy controls, with a larger coefficient for prolactinoma. This adjusted pattern differed from the direct pairwise comparisons and may reflect the influence of covariate adjustment in this relatively small exploratory cohort. Age, sex, serum prolactin, and hemoglobin were not independent predictors of FGF-23 concentrations. In particular, the loss of the prolactin association after adjustment suggests that the pooled prolactin–FGF-23 correlation may have been influenced by between-group differences rather than representing a consistent within-group relationship. In contrast, the overall α-Klotho model was not statistically significant, and none of the evaluated variables remained independently associated with α-Klotho concentrations. These findings support a cautious interpretation and require confirmation in larger prospective studies.

This study has several limitations. First, this was a single-center, cross-sectional study, and participants were recruited consecutively from a single tertiary referral center, which may have introduced selection bias and limited the generalizability of the findings. Therefore, causal relationships between FGF-23, α-Klotho, and prolactinoma cannot be established. Second, the sample size was relatively limited, particularly in the non-tumoral hyperprolactinemia group. Although the post hoc sensitivity analysis indicated adequate statistical sensitivity for the multivariable regression model, the relatively small cohort may have limited the detection of smaller effect sizes and the generalizability of the findings. Therefore, the findings require confirmation in larger, prospectively powered cohorts. Third, all patients in the prolactinoma group were receiving dopamine agonist therapy for at least six months; however, detailed treatment duration and dose-related analyses were not available. In addition, no treatment-naïve patients were included, making it difficult to distinguish the effects of prolactinoma itself from those of dopamine agonist therapy. Consequently, the observed biomarker pattern cannot be attributed exclusively to tumor biology or treatment-related effects. Although the sex distribution did not differ significantly among the study groups, female participants were overrepresented in the overall study population, reflecting the well-established epidemiology of prolactinoma, which occurs more frequently in women of reproductive age. Furthermore, an additional subgroup analysis demonstrated no significant difference in circulating FGF-23 concentrations between female and male participants. Nevertheless, the relatively small number of male participants may have limited the statistical power to detect subtle sex-related differences, and therefore the influence of sex on circulating FGF-23 and α-Klotho concentrations cannot be completely excluded. This imbalance may also limit the generalizability of the findings. Although calcium and phosphorus levels were evaluated and did not differ significantly among the groups, the lack of additional parameters related to the mineral and somatotropic axes, such as parathyroid hormone (PTH), 25-hydroxyvitamin D [25(OH)D], growth hormone (GH), and insulin-like growth factor-1 (IGF-1), limits mechanistic interpretation. However, this should also be considered in light of the absence of a significant relationship between soluble α-Klotho and calcium–mineral parameters reported by Schweizer et al. [18]. Finally, direct comparison of α-Klotho levels with other studies may be affected by differences between commercial ELISA systems. Moreover, the multivariable regression analyses and the combined ROC model should be considered exploratory because they were internally, but not externally, validated. In addition, the lack of longitudinal follow-up prevented evaluation of temporal changes in circulating FGF-23 and α-Klotho concentrations and their response to treatment over time. Therefore, the discriminatory findings should be interpreted as hypothesis-generating biomarker results rather than as a clinically ready diagnostic model.

## 5. Conclusions

In this study, dopamine agonist-treated prolactinoma was characterized by higher circulating FGF-23 concentrations than those in healthy controls and higher α-Klotho concentrations than those in both healthy controls and patients with non-tumoral hyperprolactinemia. The FGF-23 difference between prolactinoma and non-tumoral hyperprolactinemia was not statistically significant. Exploratory ROC analyses suggested moderate discriminatory performance for the individual biomarkers and higher apparent performance for the combined model; however, the combined-model performance was attenuated after leave-one-out cross-validation and has not been externally validated. The adjusted FGF-23 findings and the non-significant multivariable model for α-Klotho further support a cautious, hypothesis-generating interpretation. The observed biomarker pattern may reflect prolactinoma-related biology, dopamine agonist exposure, or both. Future prospective studies in larger, treatment-naïve cohorts, with simultaneous assessment of the GH/IGF-1 axis and treatment exposure, are required to clarify the clinical relevance of this axis.

## Figures and Tables

**Table 1:** Demographic characteristics of the study groups.

Variable	Non-Tumoral Hyperprolactinemia (*n* = 14)	Prolactinoma (*n* = 29)	Control (*n* = 30)	*p* Value
Sex, n (%)				0.177
Male	1 (7.1)	8 (27.6)	10 (33.3)	
Female	13 (92.9)	21 (72.4)	20 (66.7)	
Age (years)	24.5 (19.0–50.0) ^a^	37.0 (20.0–60.0) ^b^	22.5 (18.0–59.0) ^a^	<0.001
BMI (kg/m^2^)	22.43 (17.58–28.34)	25.71 (15.82–35.71)	23.61 (17.78–33.33)	0.080
Smoking status, n (%)				0.655
No	12 (85.7)	27 (93.1)	28 (93.3)	
Yes	2 (14.3)	2 (6.9)	2 (6.7)	

Values are presented as median (minimum–maximum) for continuous variables and as *n* (%) for categorical variables. Continuous variables were compared using the Kruskal–Wallis test. When the overall test was significant, Dunn–Bonferroni post hoc pairwise comparisons were performed. Categorical variables were compared using the appropriate exact test, as specified in Section 2.5. Superscript letters (a, b) indicate the results of Dunn–Bonferroni post hoc pairwise comparisons; groups sharing the same superscript letter are not significantly different, whereas groups with different superscript letters differ significantly. BMI: body mass index.

**Table 2:** Routine biochemical and hematological parameters of the study groups.

Variable	Non-Tumoral Hyperprolactinemia (*n* = 14)	Prolactinoma (*n* = 29)	Control (*n* = 30)	*p* Value
Glucose (mg/dL)	91.50 (82.00–101.00)	85.00 (71.00–119.00)	89.00 (74.00–99.00)	0.190
AST (U/L)	19.00 (13.00–34.00)	20.00 (12.00–32.00)	20.00 (12.00–43.00)	0.723
ALT (U/L)	16.00 (9.00–75.00)	13.00 (8.00–51.00)	21.00 (5.00–70.00)	0.405
ALP (U/L)	63.50 (57.00–320.00)	64.50 (38.00–215.00)	77.00 (46.00–198.00)	0.346
GGT (U/L)	14.00 (8.00–64.00)	11.00 (5.00–42.00)	12.00 (4.00–65.00)	0.580
LDH (U/L)	182.50 (136.00–264.00)	209.00 (124.00–406.00)	173.00 (120.00–259.00)	0.355
Total cholesterol (mg/dL)	158.50 (140.00–175.00)	173.00 (127.00–225.00)	162.00 (108.00–246.00)	0.187
LDL (mg/dL)	96.50 (53.00–115.00)	97.00 (52.00–166.00)	90.50 (20.00–148.00)	0.882
HDL (mg/dL)	42.00 (32.00–88.00)	47.50 (21.00–79.00)	47.50 (25.00–81.00)	0.642
CRP (mg/L)	3.11 (3.11–8.64)	3.14 (3.06–18.30)	3.34 (3.02–39.00)	0.758
Calcium (mg/dL)	9.55 (9.12–9.90)	9.60 (8.51–10.50)	9.50 (8.80–10.20)	0.613
Phosphorus (mg/dL)	3.55 (2.40–4.10)	3.50 (2.50–4.60)	3.30 (2.70–3.70)	0.225
Urea (mg/dL)	25.50 (13.00–34.00)	27.00 (16.00–88.00)	26.00 (16.00–43.00)	0.514
Creatinine (mg/dL)	0.70 (0.55–0.89)	0.70 (0.53–1.78)	0.75 (0.50–1.08)	0.275
Uric acid (mg/dL)	4.20 (3.00–7.90)	4.50 (2.10–17.10)	4.00 (2.90–6.40)	0.455
CK (U/L)	77.00 (11.00–583.00)	76.00 (25.00–348.00)	93.00 (32.00–183.00)	0.258
CK-MB (U/L)	15.95 (14.30–35.00)	16.55 (10.18–44.80)	23.00 (13.60–48.00)	0.362
WBC (10^3^/µL)	7.40 (4.36–10.97)	6.86 (5.19–9.51)	7.14 (3.35–10.00)	0.966
Hemoglobin (g/dL)	13.26 ± 1.11	13.21 ± 1.62	14.19 ± 2.05	0.072
Total protein (g/dL)	7.25 (6.16–7.90)	7.20 (6.10–8.30)	7.35 (6.80–8.80)	0.641
Albumin (g/dL)	4.50 (4.11–5.10)	4.65 (3.88–5.20)	4.80 (4.30–5.50)	0.053
Total bilirubin (mg/dL)	0.60 (0.40–1.50)	0.50 (0.20–1.24)	0.50 (0.30–1.20)	0.317

Values are presented as median (minimum–maximum) for non-normally distributed variables and as mean ± standard deviation (SD) for hemoglobin. The Kruskal–Wallis test was used for non-normally distributed variables, whereas hemoglobin was analyzed using one-way analysis of variance (ANOVA). No post hoc comparisons were performed for hemoglobin because the overall ANOVA was not statistically significant. ALP: alkaline phosphatase; ALT: alanine aminotransferase; AST: aspartate aminotransferase; CK: creatine kinase; CK-MB: creatine kinase-MB; CRP: C-reactive protein; GGT: gamma-glutamyl transferase; HDL: high-density lipoprotein; LDH: lactate dehydrogenase; LDL: low-density lipoprotein; WBC: white blood cell.

**Table 3:** Serum FGF-23, α-Klotho, and prolactin levels according to the study groups.

Variable	Non-Tumoral Hyperprolactinemia (*n* = 14)	Prolactinoma (*n* = 29)	Control (*n* = 30)	*p* Value
FGF-23 (pg/mL)	166.20 (100.30–327.20) ^a^	236.10 (107.70–667.90) ^b^	138.75 (49.40–253.60) ^a^	<0.001
α-Klotho (ng/mL)	2.244 (1.432–5.139) ^a^	2.908 (1.376–6.257) ^b^	2.204 (0.149–4.917) ^a^	0.001
Prolactin (ng/mL)	34.90 (21.70–59.00) ^a^	48.80 (7.30–283.00) ^a^	8.60 (1.50–18.20) ^b^	<0.001

Values are presented as median (minimum–maximum). Overall between-group comparisons were performed using the Kruskal–Wallis test. When the overall Kruskal–Wallis test was significant, Dunn–Bonferroni post hoc pairwise comparisons were performed. Superscript letters (a, b) indicate the results of Dunn–Bonferroni post hoc pairwise comparisons; groups sharing the same superscript letter are not significantly different, whereas groups with different superscript letters differ significantly. FGF-23: fibroblast growth factor-23.

**Table 4:** Correlation analysis of FGF-23 and α-Klotho levels with selected biochemical, mineral, and renal parameters.

Parameter	FGF-23 *r_s_*	FGF-23 *p* Value	α-Klotho *r_s_*	α-Klotho *p* Value
Prolactin	0.481	<0.001	0.269	0.021
Hemoglobin	−0.257	0.028	−0.234	0.046
Glucose	−0.038	0.751	−0.163	0.169
Calcium	−0.136	0.251	0.026	0.825
Phosphorus	0.036	0.761	0.004	0.973
Urea	0.219	0.063	0.029	0.808
Creatinine	−0.100	0.399	−0.135	0.256
α-Klotho	0.210	0.074	—	—

Values are Spearman correlation coefficients (*r_s_*) and corresponding *p* values. FGF-23: fibroblast growth factor-23.

**Table 5:** Univariable and multivariable linear regression analyses of factors associated with log-transformed serum FGF-23 concentrations.

Variable	Univariable B (95% CI)	*p*	Multivariable B (95% CI)	*p*
Age	0.005 (0.000–0.010)	0.058	−0.001 (−0.006–0.004)	0.661
Sex (Female vs. Male)	0.015 (−0.126–0.156)	0.835	0.132 (−0.032–0.296)	0.114
Prolactin	0.002 (0.001–0.003)	0.001	−0.00003 (−0.001–0.001)	0.962
Hemoglobin	−0.036 (−0.070 to −0.002)	0.039	−0.037 (−0.077–0.003)	0.072
Non-tumoral hyperprolactinemia (vs. Control)	0.006 (−0.151–0.163)	0.940	0.179 (0.031–0.327)	0.018
Prolactinoma (vs. Control)	0.290 (0.183–0.396)	<0.001	0.331 (0.188–0.474)	<0.001

Log-transformed serum FGF-23 concentration was used as the dependent variable. Variables with potential clinical relevance were entered into the multivariable model. The multivariable regression model was statistically significant (R^2^ = 0.391, adjusted R^2^ = 0.336; F(6,66) = 7.069; *p* < 0.001). No evidence of multicollinearity was detected (all variance inflation factor [VIF] values < 5). CI, confidence interval; FGF-23, fibroblast growth factor-23.

**Table 6:** Univariable and multivariable linear regression analyses of factors associated with log-transformed serum α-Klotho concentrations.

Variable	Univariable B (95% CI)	*p* Value	Multivariable B	*p* Value
Age	0.004 (0.000–0.009)	0.050	0.002 (−0.003–0.007)	0.483
Sex (Female vs. Male)	0.012 (−0.108–0.132)	0.844	0.063 (−0.104–0.229)	0.454
Prolactin	0.001 (0.000–0.002)	0.029	0.000078 (−0.001–0.001)	0.904
Hemoglobin	−0.018 (−0.047–0.012)	0.232	−0.017 (−0.058–0.023)	0.402
Non-tumoral hyperprolactinemia (vs. Control)	−0.015 (−0.148–0.119)	0.829	0.062 (−0.088–0.211)	0.414
Prolactinoma (vs. Control)	0.145 (0.043–0.247)	0.006	0.129 (−0.016–0.273)	0.081

Log-transformed serum α-Klotho concentration was used as the dependent variable. Variables with potential clinical relevance were entered into the multivariable model. The multivariable regression model was not statistically significant (R^2^ = 0.136, adjusted R^2^ = 0.057; F(6,66) = 1.730; *p* = 0.128). No evidence of multicollinearity was detected (all variance inflation factor [VIF] values < 5). CI, confidence interval.

**Table 7:** ROC curve analysis of FGF-23 and α-Klotho for distinguishing prolactinoma from non-tumoral hyperprolactinemia and healthy controls.

Comparison	Marker/Model	AUC	LOOCV AUC	95% CI	*p* Value	Cut-Off Value	Sens (%)	Spec (%)	Youden Index
A: Prolactinoma vs. non-tumoral hyperprolactinemia									
	FGF-23	0.741	—	0.577–0.882	0.012	183.40 pg/mL	69.0	78.6	0.475
	α-Klotho	0.732	—	0.540–0.893	0.015	2.28 ng/mL	89.7	57.1	0.468
	Combined model *	0.862	0.764	0.641–0.974	—	Model probability	82.8	92.9	0.757
B: Prolactinoma vs. Healthy Controls									
	FGF-23	0.832	—	0.728–0.922	<0.001	205.70 pg/mL	58.6	90.0	0.486
	α-Klotho	0.774	—	0.638–0.891	<0.001	2.41 ng/mL	86.2	66.7	0.529
	Combined model *	0.884	0.836	0.796–0.960	—	Model probability	89.7	73.3	0.630

ROC: receiver operating characteristic; AUC: area under the curve; CI: confidence interval; Sens: sensitivity; Spec: specificity; FGF-23: fibroblast growth factor-23. Cut-off values were determined using the maximum Youden index (J = sensitivity + specificity − 1). * The combined model was generated using an exploratory logistic regression model including both FGF-23 and α-Klotho. AUC values shown for the combined model are apparent (in-sample) estimates; the LOOCV AUC column reports the corresponding internally validated AUCs obtained by leave-one-out cross-validation (LOOCV). *p* values were not separately reported for the exploratory combined model, and because it was not externally validated, its results should be interpreted as exploratory.

## Data Availability

The raw data supporting the conclusions of this article will be made available by the authors on request.

## References

[1] Daly A.F., Beckers A. (2020). The epidemiology of pituitary adenomas. Endocrinol. Metab. Clin. N. Am..

[2] Petersenn S., Fleseriu M., Casanueva F.F., Giustina A., Biermasz N., Biller B.M.K., Bronstein M., Chanson P., Fukuoka H., Gadelha M. (2023). Diagnosis and management of prolactin-secreting pituitary adenomas: A Pituitary Society international Consensus Statement. Nat. Rev. Endocrinol..

[3] Andereggen L., Frey J., Andres R.H., Luedi M.M., Widmer H.R., Beck J., Mariani L., Christ E. (2021). Persistent bone impairment despite long-term control of hyperprolactinemia and hypogonadism in men and women with prolactinomas. Sci. Rep..

[4] Berinder K., Nyström T., Höybye C., Hall K., Hulting A.L. (2011). Insulin sensitivity and lipid profile in prolactinoma patients before and after normalization of prolactin by dopamine agonist therapy. Pituitary.

[5] Byberg S., Futtrup J., Andreassen M., Krogh J. (2019). Metabolic effects of dopamine agonists in patients with prolactinomas: A systematic review and meta-analysis. Endocr. Connect..

[6] Quarles L.D. (2012). Skeletal secretion of FGF-23 regulates phosphate and vitamin D metabolism. Nat. Rev. Endocrinol..

[7] Rausch S., Föller M. (2022). The regulation of FGF23 under physiological and pathophysiological conditions. Pflüg. Arch.-Eur. J. Physiol..

[8] Urakawa I., Yamazaki Y., Shimada T., Iijima K., Hasegawa H., Okawa K., Fujita T., Fukumoto S., Yamashita T. (2006). Klotho converts canonical FGF receptor into a specific receptor for FGF23. Nature.

[9] Kurosu H., Ogawa Y., Miyoshi M., Yamamoto M., Nandi A., Rosenblatt K.P., Baum M.G., Schiavi S., Hu M.C., Moe O.W. (2006). Regulation of fibroblast growth factor-23 signaling by Klotho. J. Biol. Chem..

[10] Quarles L.D. (2019). Fibroblast growth factor 23 and α-Klotho co-dependent and independent functions. Curr. Opin. Nephrol. Hypertens..

[11] Landry T., Shookster D., Huang H. (2021). Circulating α-Klotho regulates metabolism via distinct central and peripheral mechanisms. Metabolism.

[12] Kuro-o M., Matsumura Y., Aizawa H., Kawaguchi H., Suga T., Utsugi T., Ohyama Y., Kurabayashi M., Kaname T., Kume E. (1997). Mutation of the mouse klotho gene leads to a syndrome resembling ageing. Nature.

[13] Yamazaki Y., Imura A., Urakawa I., Shimada T., Murakami J., Aono Y., Hasegawa H., Yamashita T., Nakatani K., Saito Y. (2010). Establishment of sandwich ELISA for soluble alpha-Klotho measurement: Age-dependent change of soluble alpha-Klotho levels in healthy subjects. Biochem. Biophys. Res. Commun..

[14] Espuch-Oliver A., Vázquez-Lorente H., Jurado-Fasoli L., de Haro-Muñoz T., Díaz-Alberola I., López-Velez M.D.S., de Haro-Romero T., Castillo M.J., Amaro-Gahete F.J. (2022). Reference values of soluble α-Klotho serum levels using an enzyme-linked immunosorbent assay in healthy adults aged 18–85 years. J. Clin. Med..

[15] Brobey R.K., German D., Sonsalla P.K., Gurnani P., Pastor J., Hsieh C.C., Papaconstantinou J., Foster P.P., Kuro-o M., Rosenblatt K.P. (2015). Klotho protects dopaminergic neuron oxidant-induced degeneration by modulating ASK1 and p38 MAPK signaling pathways. PLoS ONE.

[16] Melmed S., Casanueva F.F., Hoffman A.R., Kleinberg D.L., Montori V.M., Schlechte J.A., Wass J.A.H. (2011). Diagnosis and treatment of hyperprolactinemia: An Endocrine Society Clinical Practice Guideline. J. Clin. Endocrinol. Metab..

[17] Arslan M.S., Sahin M., Karakose M., Tutal E., Topaloglu O., Ucan B., Demirci T., Caliskan M., Ozdemir S., Ozbek M. (2017). Serum levels of fibroblast growth factor-23, osteoprotegerin, and receptor activator of nuclear factor kappa B ligand in patients with prolactinoma. Endocr. Pract..

[18] Schweizer J.R.O.L., Schilbach K., Haenelt M., Gagliardo A., Peters A., Thorand B., Störmann S., Schopohl J., Reincke M., Lauseker M. (2025). Soluble alpha klotho—Impact of biological variables and reference intervals for adults. Eur. J. Endocrinol..

[19] Arslan I.E., Ozturk B.O., Bolayir B., Yalcin M.M., Yetkin I., Akturk M. (2022). Circulating kisspeptin and klotho levels in women with hyperprolactinemia. Turk. J. Endocrinol. Metab..

[20] Rudman Y., Duskin-Bitan H., Richter I., Tsvetov G., Masri-Iraqi H., Akirov A., Shimon I. (2023). Hemoglobin decline as a signal for hyperprolactinemia onset prior to prolactinoma diagnosis in hypogonadal men. Andrology.

[21] Odaka H., Numakawa T., Adachi N., Ooshima Y., Nakajima S., Katanuma Y., Inoue T., Kunugi H. (2014). Cabergoline, dopamine D2 receptor agonist, prevents neuronal cell death under oxidative stress via reducing excitotoxicity. PLoS ONE.

